# *Aphanius arakensis*, a new species of tooth-carp (Actinopterygii, Cyprinodontidae) from the endorheic Namak Lake basin in Iran

**DOI:** 10.3897/zookeys.215.1731

**Published:** 2012-08-17

**Authors:** Azad Teimori, Hamid Reza Esmaeili, Zeinab Gholami, Neda Zarei, Bettina Reichenbacher

**Affiliations:** 1 Department of Earth- and Environmental Sciences, Palaeontology & Geobiology & GeoBio-CenterLMU, Ludwig-Maximilians-University, Richard-Wagner-Strasse 10, D-80333 Munich, Germany; 2 Department of Biology, College of Sciences, Shiraz University, Shiraz 71454, Iran; 3 Department of Biology, Faculty of Sciences, Shahid-Bahonar University of Kerman 22 Bahman Blvd. Kerman, 76169-14111 Iran

**Keywords:** Male color patterm, freshwater fish, tooth-carp, biodiversity, evolution, sexual selection

## Abstract

A new species of tooth-carp, *Aphanius arakensis* **sp. n**., is described from the Namak Lake basin in Iran. The new species is distinguished by the congeners distributed in Iran by the following combination of characters: 10–12 anal fin rays, 28–32 lateral line scales, 10–13 caudal peduncle scales, 8–10 gill rakers, 12–19, commonly 15–16, clearly defined flank bars in males, a more prominent pigmentation along the flank added by relatively big blotches in the middle and posterior flank segments in females, a short but high antirostrum of the otolith that has a wide excisura, and a ventral rim with some small, drop-like processes, and 19 molecular apomorphies (17 transitions, two transversions) in the cytochrome *b* gene. It was suggested based on the phylogenetic analysis that the new species is sister to *Aphanius sophiae* from the Kor River and that *Aphanius farsicus* from the Maharlu Lake basin is sister to *Aphanius arakensis* plus *Aphanius sophiae*. A noticeable feature of the *Aphanius* diversity in Iran is the conservatism of the external morphology as well as morphometric and meristic characters, while distinctive differences are present in genetic characters, otolith morphology, and male color pattern. Transformation of the latter was probably driven by sexual selection.

## Introduction

*Aphanius* is the only representative of the Cyprinodontidae (Teleostei, Cyprinodontiformes) in Eurasia. The genus occurs in coastal (brackish) and landlocked (freshwater to saline) water bodies in the Mediterranean and Persian Gulf basins from Iberian Peninsula as far eastwards as Iran and Pakistan (Wildekamp 1993). *Aphanius* species diversity is highest in the endorheic basins of the mountainous regions of central Anatolia and the Iranian plateau (Coad 2000; Hrbek and Meyer 2003, Hrbek et al. 2006, Esmaeili et al. 2012). Though central Anatolia is believed to represent the center of *Aphanius* speciation (Wildekamp et al. 1999), a high number of *Aphanius* species also occurs in Iran. Apart from the widely distributed *Aphanius dispar* (Rüppell, 1829), seven endemic *Aphanius* species have been described from Iran to date, namely *Aphanius ginaonis* (Holly, 1929) from the Genow hot spring near the Persian Gulf; *Aphanius isfahanensis* Hrbek, Keivany & Coad, 2006 from the endorheic Esfahan basin; *Aphanius farsicus* Teimori, Esmaeili and Reichenbacher, 2011 from the endorheic Maharlu Lake basin [*Aphanius farsicus* is a replacement name for the previous *Aphanius persicus* (Jenkins, 1910) because this name has been recognized as a homonym of the fossil *Aphanius persicus* (Priem, 1908) (Gaudant 2011, Teimori et al. 2011)]; *Aphanius sophiae* (Heckel, 1849) from the endorheic Kor River Basin; *Aphanius vladykovi* Coad, 1988 from the upper reaches of the Karoun basin; *Aphanius mesopotamicus* Coad, 2009 from the Tigris-Euphrates drainage; and the recently re-established *Aphanius pluristriatus* (Jenkins, 1910) from the Mond River drainage. In addition to the species listed above, *Lebias punctatus* and *Lebias crystallodon* were originally described from the Nemek Deria near Shiraz by Heckel (1846–1849). Berg (1949) and Coad (1996) considered *Lebias punctatus* to be a synonym of *Aphanius sophiae* but at that time most of now valid species distributed in Iran were thought to be synonyms of the widely distributed *Aphanius sophiae*. Coad (1996) strongly suggested that the type locality of *Lebias punctatus* is not the Lake Maharlu but some other lake nearby as a name Nemek Deria is a very common name in Farsi for a salt lake. However, later, the Kotschy’s itinerary in southern Iran in 1841 and 1842 was studied in detail based on botanical labels and it was clearly shown that collections by Kotschy studied by Heckel indeed came from a lake now called Maharlu (Edmondson and Lack 2006). This aspect is not in the focus of this very paper; we tentatively consider *Lebias punctatus* to be a synonym of *Aphanius sophiae* until a proper examination of the extant syntypes of *Lebias punctatus* is done.

**Figure 1.**
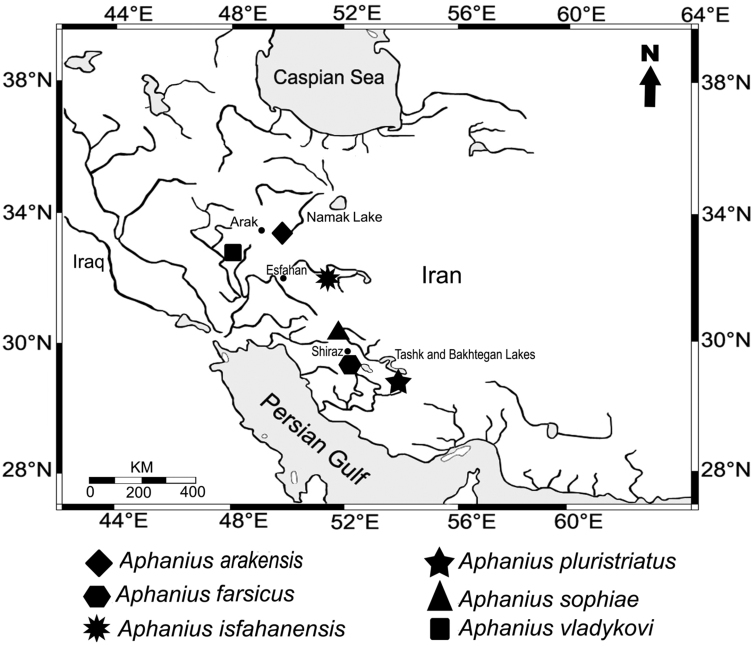
Geographic distribution of the endemic Iranian inland *Aphanius* species.

A number of isolated *Aphanius* populations that might deserve species status have been reported from endorheic drainages in Iran, but have not yet been investigated in detail (Coad and Abdoli 2000; Hrbek et al. 2006; Esmaeili et al. 2010). They were commonly identified as *Aphanius sophiae* (Heckel, 1849) (Coad and Abdoli 2000; Kamal et al. 2009); however, it was shown that the true *Aphanius sophiae* is restricted to the endorheic Kor River basin near Shiraz (Fars Province) (Coad 2009; Esmaeili et al. 2012). This study describes a newly discovered *Aphanius* population from the Namak Lake basin in northern central Iran (Fig. 1). The specimens were collected in 2007 because they appeared to be different from other Iranian *Aphanius* species by a specific coloration. Here it is shown that the population from the Namak Lake basin in fact represents a new species, *Aphanius arakensis*. Our study is based on a total-evidence approach including morphometric and meristic characters, otolith morphology, and molecular data.

## Material and methods

***Institutional acronyms*:** ZM-CBSU, Zoological Museum of Shiraz University, Collection of Biology Department; ZSM, Zoological State Collection, Munich.

***Material for morphological comparison***

*Aphanius sophiae*: 35 males (19.3–33.3 mm SL) and 35 females (18.6–36.6 SL) from the Ghadamgah spring-stream system (close to type locality) in the Kor River Basin (Iran, Fars Province), 30°15'N, 52°25'E. Males: ZM-CBSU, 8460, 8462, 8462, 8466, 8468, 8470, 8472-73, 8475, 8477, 8479, 8481, 8483, 8485, 8487, 8479, 8489, 8491-97, 8499, 8501, 8503-09, 8511-13; females: ZM-CBSU, 8461, 8463, 8465, 8467, 8469, 8471, 8474, 8476, 8478, 8480, 8482, 8484, 8486, 8488, 8490, 8498, 8500, 8502, 8510, 8514-29.

*Aphanius farsicus*: 35 males (20.0–26.8 mm SL) and 35 females (20.4–35.2 mm SL) from the Barm-e-Shur spring in the Maharlu Lake Basin (type locality) (Iran, Fars Province), 29°27'N, 52°42'E. Males: ZM-CBSU, 9413, 9415, 9417, 9421, 9441, 9443, 9447, 9449, 9459, 9467, 9481, 9483, 9485, 9487, 9489, 9493, 9497, 9499, 9503, 9511, 9513, 9515, 9517, 9519, 9527, 9529, 9531, 9533, 9537, 9539, 9541, 9555, 9557, 9559, 6375; females: ZM-CBSU, 9410, 9412, 9420, 9422, 9428, 9442, 9444, 9452, 9458, 9472, 9474, 9478, 9482, 9488, 9492, 9494, 9498, 9500, 9502, 9504, 9506, 9510, 9516, 9520, 9530, 9532, 9534, 9536, 9558, 9560, 9562, 9564, 6364, 6359, 6385.

*Aphanius isfahanensis*: 18 males (17.6–23.8 mm SL) and 25 females (17.7–34.0 mm SL) from the Zayanderh River near Varzaneh, Esfahan Basin (type locality) (Iran, Esfahan Province), 32°25'N, 52°39'E. Males: ZM-CBSU, 6472, 6474, 6476, 6478, 6480, 6482, 6484, 6486, 6488, 6490, 6492, 6494, 6496, 6498, 6500, 8602, 8604, 8613; females: ZM-CBSU, 6471, 6473, 6475, 6477, 6479, 6481, 6483, 6485, 6487, 6489, 6491, 6493, 6495, 6497, 6499 6501, 8603, 8605-8612.

*Aphanius vladykovi*: 35 males (17.3–29.2 mm SL) and 35 females (16.1–41.4 mm SL) from the Chaghakhor wetland in the upper reaches of the Karoun Basin (Iran, Chahar Mahale Bakhtyari Province), 31°55'N, 50°56'E. Males: ZM-CBSU, 6408-09, 6413-14, 6416, 6418, 6420-21, 6423, 6425-27, 6430, 6433-41, 6443-44, 6446, 6448-49, 6451-57: females: ZM-CBSU, 6401-03, 6405-07, 6410-12, 6415, 6417, 6419, 6422, 6424, 6428-29, 6431-32, 6442, 6445, 6447, 6450, 6458-70.

***Material for molecular comparison***

*Aphanius sophiae* ZM-CBSU, M46, M97, M98, M174-176 (Ghadamgah spring-stream system); *Aphanius farsicus* ZM-CBSU, M47, M136, M177-178 (Barm-e-Shur spring); *Aphanius isfahanensis* ZM-CBSU, M211, M213-214 (Zayanderh River near Varzaneh); *Aphanius arakensis* sp. n. ZM-CBSU, M198-200 (Namak Lake Basin, 34°00'N, 49°50'E); *Aphanius vladykovi* ZM-CBSU, M60, M139, M209 (Chaghakhor wetland in the upper reaches of the Karoun Basin).

***Materials from GenBank*.** *Aphanius vladykovi*: GenBank DQ367526; *Aphanius fasciatus*:GenBank AF299273; *Aphanius iberus*:GenBank AF299290. *Poeciliopsis gracilis* (GenBank AF412155) was used as outgroup.

### Morphological analysis

Based on the morphometric schemes introduced in Holcik et al. (1989) and Doadrio et al. (2002), 18 morphometric parameters were measured using a Vernier calliper and recorded to the nearest 0.5 mm. The standard length was measured from the most anterior part of the snout to the base of the caudal fin rays. In total, 21 relative variables were calculated from the measurements (Table 1).

Scales removed from the left side of each fish, from the 3rd or 4th row below the dorsal fin, were mounted between microscope slides, and length and width of scales were measured to the nearest 0.1 mm by using a scale reader (Xerox 320). For each individual, scale length and scale width measurements were averaged to obtain a single length value and a single width value per individual and relative width and length of scales were calculated following Esmaeili (2001).

The meristic characters were counted under a stereomicroscope and consist of the numbers of (i) dorsal (ii) pectoral (iii) pelvic and (vi) anal fin rays, (v) lateral line series scales, (vi) caudal peduncle scales (the numbers of scales along the caudal peduncle, i.e. from the base of the last anal fin ray to the base of the caudal fin rays in a direct line), (vii) gill rakers and (viii) flank bars of males. Two posteriormost rays in dorsal and anal fins were calculated as one ray.

For examination of otolith morphology fish skulls were opened ventrally in order to remove the right and left otoliths. Otoliths were cleaned from tissue remains in 1% potassium hydroxide solution for 3–6 h, washed several times and finally rinsed in distilled water for 12 h. Otolith morphology was analyzed under a stereo microscope. In addition, five or six otoliths from each population were examined by a scanning electron microscope (SEM) with a LEO 1430 VP at ZSM.

Univariate analysis of variance (ANOVA, with Duncan’s post hoc test, p < 0.05) was used to test the significance of phenotypic differences among species and also between sexes. Canonical discriminant analysis (CDA) was used for multivariate analyses in order to document the classification success of the groups. The statistical analyses were carried out using PASW 19.00 (SPSS Inc 2011) and PAST (Hammer et al. 2001: PAlaeontological STatistics, version 1.81).

### Laboratory protocols and molecular analyses

Total genomic DNA was extracted according to phenol/chloroform procedures (Sambrook et al. 1989). A 900 base pairs (bp) fragment of the cytochrome b gene was successfully amplified via PCR using the primers (forward: Glu-F, 5’ - AACCACCGTTGTATTCAACTACAA-3’; reverse: ThrR, 5’-CCTCCGATCTTCGGATTACAAGACCG-3’ (Machordom and Doadrio 2001). Ampliﬁcation was performed in a thermal cycler programmed as follows: initial 94 °C for 3 min, 35 cycles at 94 °C for 50 s, 56 °C for 45s, 72 °C for 1 min, followed by a ﬁnal extension at 72 °C for 5 min. Sequencing was performed by Macrogen company, South Korea. Cytochrome *b* nucleotide sequences were edited with BioEdit and aligned through Geneious pro v5.4 (Drummond et al. 2011). Additional *Aphanius* sequences were obtained from the NCBI GenBank (http://www.ncbi.nlm.nih.gov) and included in the analyses (see above). The achieved cytb sequences for the here studied *Aphanius* populations were deposited in GenBank under numbers JX154880–JX154898.

Maximum likelihood-based phylogenetic relationships were estimated by using the program SeaView version 4 (Gouy et al. 2010). The best-fit model of nucleotide substitution was obtained using the program JmodelTest 0.1.1 (Posada 2008). Accordingly, the GTR + I + G model (= General Time Reversible model + proportion of Invariable sites + Gamma-shaped distribution of rates across sites) was chosen.

Maximum parsimony based phylogenetic relationships were estimated using the program SeaView version 4 (Gouy et al. 2010) with 100 heuristic searches using random additions of sequences and implementing the Close-Neighbor-Interchange (CNI) on random tree algorithm. To test this phylogeny, bootstrap method using 2000 replication was used. To document the degree of homoplasy and degree to which potential synapomorphy is exhibited on the tree, the Consistency Index (CI) and the Retention Index (RI) were calculated by using the parsimony model within the Mesquite system for phylogenetic computing (Maddison and Maddison 2011).

The Neighbor Joining (NJ) distance-based phylogenetic relationships were estimated by using the computer program Geneious pro v5.4 (Drummond et al. 2011). The HKY85 model (Hasegawa et al. 1985) of molecular evolution was used with gamma distributed among site rate variation. There were a total of 771 positions in the final dataset.

**Table 1.** Morphometric characters of *Aphanius arakensis* sp. n. and other Iranian *Aphanius* species. Each cell contains mean ± standard deviation and range (minimum–maximum).

**Character**	***Aphanius arakensis*n=35, male**	***Aphanius arakensis*n=35, female**	***Aphanius isfahanensis*n=18, male**	***Aphanius isfahanensis*n=25, female**	***Aphanius sophiae*n=35, male**	***Aphanius sophiae*n=35, female**	***Aphanius farsicus*n=35, male**	***Aphanius farsicus*n=35, female**	***Aphanius vladykovi*n=35, male**	***Aphanius vladykovi*n=35, female**	***Aphanius pluristriatus*n=32, male**	***Aphanius pluristriatus*n=38, female**
**% Standard length**												
Head length	29.2±0.1(26.9–30.9)	28.5±0.9(26.8–30.3)	29.8±1.2(27.7–31.6)	29.2±1.2(27.4–32.1)	29.4±1.1(27.3–31.5)	28.0±1.3(25.4–30.6)	32.1±1.3(29.8–35.2)	31.6±1.5(28.1–34.1)	31.2±1.3(28.9–33.9)	30.9±1.4(27.3–33.6)	30.2±1.8(27.1–36.8)	28.5±1.4(29.8–35.5)
Head depth	22.1±0.8(20.0–23.9)	21.5±0.8(20.0–23.5)	20.9±1.0(18.9–22.3)	20.2±1.0(17.8–22.3)	21.9±0.8(19.3–23.9)	21.1±1.12(18.8–23.9)	23.9±1.4(20.8–26.1)	22.9±1.3(20.0–25.0)	23.7±0.9(21.9–26)	23.2±1.2(20.5–25.7)	21.6±1.1(19.7–24.5)	19.8±0.9(23.3–21.2)
Predorsal length	61.1±1.1(59.0–63.8)	61.3±1.8(58.1–67.7)	59.7±1.6(55.2–62.8)	60.1±2.3(56.7–65.8)	60.1±1.9(55.2–63.5)	61.2±1.6(57.3–83.4)	61.5±1.4(58.2–64.9)	62.5±1.1(59.4–67.1)	63.6±1.4(60.5–66.4)	63.1±1.9(59.4–67.3)	64.8±2.8(59.6–75.4)	61.0±1.7(68.7–64.2)
Length of pectoral fin	18.1±0.1(15.5–19.8)	17.5±1.2(12.9–19.3)	16.4±1.3(12.9–19.0)	16.2±1.2(13.9–18.2)	18.3±1.1(15.8–20.1)	17.0±1.3(14.9–19.6)	19.4±1.2(17.1–21.8)	17.2±1.1(15.0–19.4)	16.3±1.2(14–20.3)	15.4±1.1(13.5–17.6)	18.6±1.8(14.6–22.1)	17.8±1.3(14–20.4)
Length of pelvic fin	7.9±0.6(6.6–9)	7.9±0.5(7.1–9.3)	6.7±0.7(5.7–8.4)	6.6±0.8(5.1–7.1)	10.1±0.8(8.7–11.7)	8.7±0.8(6.9–10.3)	8.6±0.8(6.7–10.2)	7.6±0.9(5.7–9.2)	6.7±0.7(5.4–8.1)	6.5±0.8(4.5–8.0)	7.4±1.3(4.7–11.0)	6.7±0.6(5.0–8.0)
Length of anal fin	12.5±1.7(9.8–14.3)	11.7±1.2(10.4–13.4)	11.4±2.4(9.6–12.7)	10.7±2.8(8.7–13.3)	13.4±1.7(11–16.1)	11.8±1.9(14.1–11.8)	13.4±2.6(10.7–17.2)	11.8±2.1(9.7–13.3)	12.8±1.0(10.6–15.2)	11.7±2.0(9.3–16.8)	13.7±1.0(11.5–15.3)	12.5±1.1(8.9–14.5)
Minimum body depth	16.5±0.6(15.1–17.7)	15.2±0.7(13.8–16.5)	14.8±0.6(14.0–15.8)	14.4±0.8(12.4–15.6)	16.6±1.1(14.3–18.9)	14.7±0.8(13.3–16.8)	15.5±1.1(13.3–17.9)	13.8±0.9(11.8–15.9)	14.8±1.0(12.0–16.7)	13.9±1.1(11.6–16.1)	17.1±1.1(14.7–19.6)	16.1±1.1(12.6–17.1)
Pectoral - anal fins distance	37.1±1.5(34.3–39.3)	38.3±1.4(35.1–40.1)	34.6±1.6(31.9–37.6)	38.2±2.8(34.4–43.6)	35.1±1.8(31.5–40.6)	39.2±2.6(34.6–43.7)	35.7±2.7(32.17–42)	37.7±2.1(33.8–42.1)	34.7±2.1(30–39.3)	35.5±1.9(31.8–39.9)	36.8±2.4(30.1–44.8)	37.1±1.7(32.0–39.5)
Pectoral - pelvic fins distance	23.3±1.5(20.5–26.4)	23.8±1.4(20.8–26.9)	22.1±1.7(19.3–25.4)	25.2±2.8(20.7–29.6)	21.1±1.1(18.4–23.2)	23.5±2.5(18.6–28.0)	22.8±2.6(18.1–30)	24.1±2.1(19.6–28.6)	22.2±2.1(17.8–25.9)	22.7±1.6(19.5–25.9)	24.1±2.2(20.1–31.5)	23.6±1.7(18.4–26.7)
Pelvic - anal fins distance	13.1±0.8(10.8–14.5)	13.6±0.9(11.9–15.2)	11.7±1.0(9.6–13.8)	12.5±1.2(10.1–14.4)	14.1±1.1(11.8–17.6)	15.2±1.2(12.9–18.0)	13.2±1.1(11.1–15.4)	13.4±1.1(11.3–16.5)	12.1±1.2(9.9–14.4)	12.8±1.2(10.5–14.9)	13.5±0.9(11.9–16.9)	13±1.1(9.5–14.9)
Length of caudal peduncle	22.1±0.8(19.9–23.9)	21.9±0.7(20.0–23.2)	23.7±1.3(21.6–27.2)	22.8±1.1(20.0–24.4)	22.8±1.4(19.7–25.7)	23.4±1.7(19.6–26.9)	21.7±1.3(18.9–23.9)	20.8±1.4(17.9–23.6)	21.2±1.1(19.1–24.1)	21.5±1.6(17.9–25.2)	22.8±1.3(19.6–25.7)	23.1±1.4(19.6–26.3)
Length of caudal fin	20.3±1.5(14.9–23.1)	19.5±0.1(16.8–21.6)	20.2±1.9(17.4–24.3)	19.2±1.4(16.4–21.3)	20.1±1.3(17.7–23.3)	18.1±1.6(14.9–22.6)	21.7±1.0(17.8–24.6)	19.1±1.3(16.7–21.6)	20.3±1.25(17.5–22.5)	19.2±1.4(16.2–22.3)	20.4±2.3(15.8–26.4)	19.7±1.2(13.3–22.7)
Preanal distance	68.4±1.3(66.3–71.2)	69.2±0.1(66.7–71.7)	67.2±0.9(62.3–71.7)	70.5±1.0(63.6–74.6)	68.1±2.1(64.2–73.3)	69.2±1.9(65.4–73.9)	69.9±1.3(66.5–76.1)	71.2±0.9(67.2–76.6)	67.8±1.8(62.9–72.4)	67.9±1.3(63.9–73.7)	69.2±1.9(65.7–73.2)	68.1±1.4(65.4–71.5)
Scale length	3.8±0.2(3.5–4.2)	3.8±0.2(3.3–4.4)	3.1±0.2(2.7–3.5)	3.2±0.3(2.6–4.0)	3.1±0.3(2.7–3.7)	3.1±0.3(2.6–3.8)	3.5±0.4(2.7–4.5)	3.4±0.4(2.5–4.6)	2.6±0.3(1.9–3.3)	2.4±0.3(1.8–3.2)	3.9±0.2(3.4–4.5)	4.1±0.2(3.8–4.5)
Scale width	3.9±0.2(3.5–3.4)	3.8±0.2(3.3–4.6)	3.7±0.3(3.3–4.3)	3.8±0.4(3.1–4.9)	3.4±0.4(2.8–4.18)	3.3±0.4(2.6–4.1)	4.1±0.6(3.2–5.4)	3.9±0.5(3.1–4.9)	2.7±0.3(1.9–3.2)	2.5±0.4(1.8–3.2)	4.1±0.4(2.8–5.4)	4.2±0.2(3.7–4.8)
**% Preanal distance**												
Minimum body depth	24.1±0.1(21.7–25.9)	22.0±0.8(20.2–23.4)	22.0±1.3(19.7–24.5)	20.1±1.3(18.3–23.1)	24.4±1.4(21.5–27.1)	21.3±1.1(19.3–23.3)	22.2±1.4(19–25.1)	19.4±1.4(16.8–22.8)	21.9±1.6(17.6–24.8)	20.5±1.7(16.9–23.5)	24.5±1.3(21.2–27.1)	23.7±1.6(18.8–26.5)
Length of caudal peduncle	32.2±1.5(29.0–34.8)	31.7±1.2(28.6–34.0)	35.3±1.7(31.9–37.9)	32.4±2.4(27.4–38.4)	33.6±2.4(27.9–38.7)	33.9±3.1(27.2–40.3)	31.1±2.5(25.1–35.7)	29.3±2.5(23.9–33.8)	31.3±1.8(28.0–35.5)	31.6±2.7(26.1–38.1)	33.1±2.2(27.4–37.8)	33.8±2.3(28.2–38.1)
Length of caudal fin	29.6±2.3(21.5–33.5)	28.2±1.7(24.4–32.0)	30.1±3.2(25.7–36.6)	27.3±2.4(22.9–32.6)	29.4±1.1(25.4–33.8)	26.2±2.7(21.2–33.9)	31.0±2.3(26.6–35.4)	26.7±2.1(23.1–32.1)	29.9±1.9(27.0–33.6)	28.2±2.2(24.1–34.9)	29.4±3.0(23.2–36.6)	28.1±2.9(19.5–33.3)
Eye diameter	12.6±0.9(10.7–14.8)	12.0±1.19(10.5–13.8)	15.5±2.6(13.6–17.4)	13.7±1.3(11.1–15.9)	13.7±0.9(11.5–14.9)	12.7±1.1(10.0–15.3)	15.1±2.6(13.4–17)	13.5±2.2(10.6–16.1)	13.7±1.1(11.9–16.2)	14.2±1.9(11.3–15.3)	14.4±0.9(12.9–17.4)	14.1±1.6(12.4–18.1)
**% Head width**												
Interorbital distance	1.1±0.06(0.9–1.2)	1.1±0.1(0.9–1.2)	0.9±0.1(0.8–1.1)	0.9±0.06(0.76–1.1)	0.9±0.06(0.8–1.1)	0.9±0.1(0.8–1.1)	1.1±0.1(0.8–1.2)	1.1±0.1(0.8–1.2)	0.8±0.05(0.7–0.9)	0.8±0.06(0.7–0.9)	0.9±0.05(0.8–1.1)	1.0±0.06(0.8–1.1)
**% Head length**												
Eye diameter	0.3±0.01(0.2–0.3)	0.3±0.2(0.2–0.3)	0.3±0.01(0.3–0.4)	0.3±0.02(0.3–0.4)	0.3±0.02(0.3–0.35)	0.3±0.02(0.2–0.35)	0.3±0.02(0.3–0.4)	0.3±0.03(0.2–0.3)	0.3±0.02(0.2–0.3)	0.3±0.1(0.2–1.2)	0.3±0.02(0.3–0.4)	0.3±0.02(0.3–0.4)
**% Eye diameter**												
Preorbital distance	0.8±0.1(0.7–0.9)	0.8±0.1(0.7–1.0)	0.7±0.05(0.6–0.8)	0.7±0.1(0.6–1.0)	0.8±0.1(0.7–1.1)	0.8±0.1(0.6–1.0)	0.8±0.1(0.6–1.1)	0.8±0.08(0.7–1.1)	0.9±0.1(0.8–1.1)	0.9±0.1(0.2–1.1)	0.8±0.05(0.6–0.9)	0.8±0.1(0.6–0.9)
**% Minimum body depth**												
Length of caudal peduncle	1.3±0.1(1.2–1.5)	1.4±0.1(1.3–1.6)	1.6±0.1(1.5–1.9)	1.6±0.1(1.3–1.8)	1.4±0.15(1.1–1.7)	1.6±0.15(1.3–2.0)	1.4±1.5(1.1–1.8)	1.5±0.1(1.2–1.9)	1.4±0.1(1.2–1.7)	1.5±0.2(1.1–1.9)	1.3±0.1(1.2–1.6)	1.4 ±0.2(1.2–1.6)

## Results

### Aphanius arakensis sp. n.

urn:lsid:zoobank.org:act:D9995F4C-AF0A-4791-9D80-D759EFEDA569

http://species-id.net/wiki/Aphanius_arakensis

Figure 2A, B

#### Holotype.

Male, 38.5 mm TL, 31.5 mm SL, Iran, Arak, Namak Lake Basin, 34°00'N, 49°50'E, Altitude 1786 m, 26 September 2007, A. Teimori, M. Ebrahimi, A. Gholamifard and A. Gholmhosseini (ZM-CBSU 10999).

#### Paratypes.

35 males (22.6–32.7 mm SL), 35 females (22.5–34.1 mm SL), same locality as holotype (ZM-CBSU 11000, 11051–11118).

#### Diagnosis.

The new species is distinguished by the congeners distributed in Iran by the following combination of characters: 10–12 anal fin rays, 28–32 lateral line scales, 10–13 caudal peduncle scales, 8–10 gill rakers, 12–19, commonly 11–13, clearly defined flank bars in males, a more prominent pigmentation along the flank added by relatively big blotches in the middle and posterior flank segments in females, a short but high antirostrum of the otolith that has a wide excisura, and a ventral rim with some small, drop-like processes and 19 molecular apomorphies (17 transitions, two transversions) in the cytochrome *b* gene.

#### Description of the holotype.

The males of the new species reach approximately 32 mm SL and have 12–19 flank bars, the females are usually larger than the males and reach approximately 34 mm SL.

The morphometric characters are summarized in Table 1. Compared to the other examined *Aphanius* species, *Aphanius arakensis* sp. n. shows higher mean values of the minimum body depth, width and length of scales, distances between the pectoral and pelvic fins and the interorbital distance, but significantly lower mean values for the eye diameter and the caudal peduncle length (differences are statistically significant, p < 0.05).

The meristic characters are summarized in Table 2. The dorsal fin is characterized by a somewhat curved superior border, and has 11–14 rays; the anal fin shows a round superior border and includes 10–12 rays; the pectoral fin is rounded and consists of 14–18 rays; the pelvic fin is relatively short, positioned just anteriorly to the anal fin and comprises 6–8 rays. The caudal fin is rounded; the caudal peduncle possesses 10–13 scales. The number of lateral line series scales is 27–32. However, the ANOVA analysis reveals that only the numbers of lateral line series scales and caudal peduncle scales (in males and females), as well as the numbers of flank bars (in males), significantly differ from the values obtained for the other examined species. Moreover, there is a significant correlation between SL and numbers of flank bars (Pearson Correlation r = 0.455, p < 0.05*).

The otolith is rounded-trapezoid and characterized by a very wide excisura, a medium-sized and pointed rostrum, and a quite short antirostrum. The ventral and dorsal rims are slightly curved; the ventral rim may bear small irregular processes; the dorsal rim may show a fine crenulation; the posterior rim is steep (Fig. 3W-Aa).

**Figure 2.**
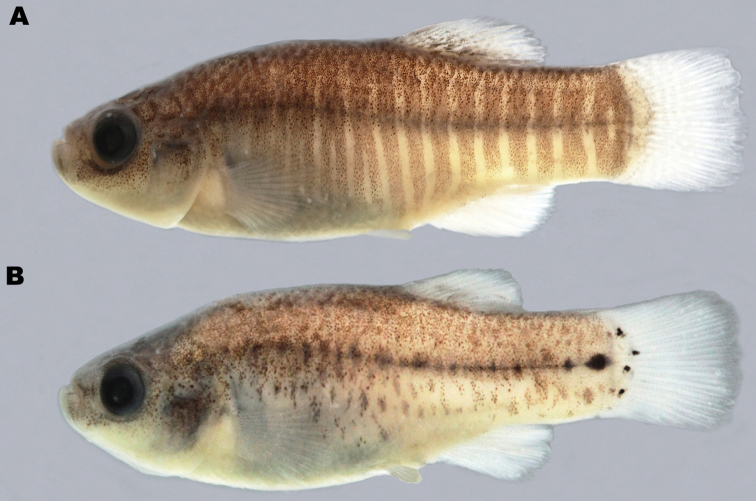
**A** *Aphanius arakensis*, holotype, male, 31.5 mm SL (ZM-CBSU 10999) **B** paratype, female, 31.5 mm SL (ZM-CBSU 11054).

The flank bars in males (Fig. 2a) are narrow and the interspaces are broader than the bars. The first bar is located above the operculum, while the posteriormost bar is located at the base of the caudal fin; the interspaces are wider at the caudal peduncle than in the anterior body part. Dorsally, the head is gray and the body is dark due to a strong melanophore pigmentation. The ventral body portion does not usually show any dark pigmentation. The dorsal, anal and caudal fins have white margins; the first rays of the dorsal fin are dark. The pectoral fins are somewhat yellowish. The pelvic fin is yellowish. Most specimens are characterized by dark blotches at the base of the dorsal and anal fins.

Females (Fig. 2b) are characterized by a grayish pigmentation of the back. The lateral flanks of the body are covered by dark pigmentations; series of blotches are present from the middle of the body to the caudal peduncle. The ventral part of the head and belly are light. The chin and sides of the head are speckled with melanophores. Below the eye there is a line of relatively dark melanophores. All fins are white.

**Figure 3.**
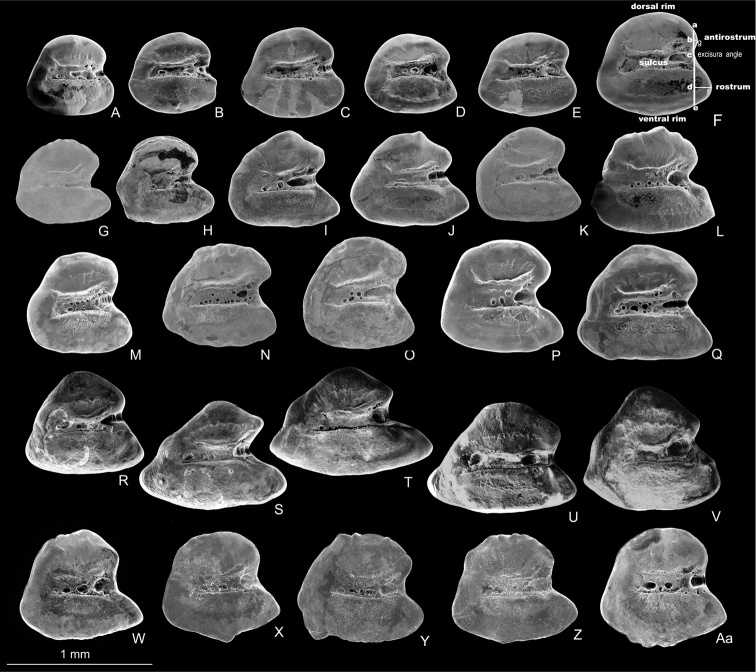
Left otoliths (medial view) of *Aphanius isfahanensis* (**A–F**), *Aphanius farsicus* (**G–L**), *Aphanius sophiae* (**M–Q**), *Aphanius vladykovi* (**R–V**) and *Aphanius arakensis* (**W–Aa**). Otolith terminology and taxonomic most informative morphometric distances are indicated in Fig. 3F and include height of antirostrum (**a–c**), height of rostrum (**c–e**), length of antirostrum (**b–g**), and length of rostrum (**d– f**). SEM pictures.

**Table 2.** Meristic characters (mean ± standard deviation and range) of Iranian *Aphanius* species.

**Character**	***Aphanius arakensis*n=35, male**	***Aphanius arakensis*n=35, female**	***Aphanius isfahanensis*n=18, male**	***Aphanius isfahanensis*n=25, female**	***Aphanius sophiae*n=35, male**	***Aphanius sophiae*n=35, female**	***Aphanius farsicus*n=35, male**	***Aphanius farsicus*n=35, female**	***Aphanius vladykovi*n=35, male**	***Aphanius vladykovi*n=35, female**	***Aphanius pluristriatus*n=32, male**	***Aphanius pluristriatus*n=38, female**
Dorsal fin rays	12.2±0.8(11–14)	12.3±0.7(11–14)	11.7±0.6(11–13)	11.6±0.7(10–13)	13.7±0.6(13–15)	13.8±0.8(13–15)	12.1±0.8(11–14)	12±0.8(10–13)	13.2±0.8(12–15)	13.5±0.7(12–15)	13.8±0.7(12–15)	13.6±0.7(12–15)
Pectoral fin rays	16.7±0.9(14–18)	16.8±0.6(16–18)	15.9±0.8(15–17)	16.3±0.5(15–17)	18±0.8(16.20)	17.9±0.7(17–19)	15.4±0.7(14–17)	15.4±0.6(14–17)	16.5±0.8(14–18)	16.6±0.7(15–18)	17.1±0.7(16–19)	17±0.6(16–18)
Pelvic fin rays	7.3±0.5(6–8)	7.2±0.4(7–8)	7±0.6(6–8)	6.9±0.5(6–8)	7.3±0.5(6–8)	7.3±0.6(6–8)	6.7±0.4(6–7)	6.6±0.5(5–7)	7±0.6(6–9)	6.9±0.4(6–8)	7.2 ±0.5(6–8)	7.1± 0.4(8–7)
Anal fin rays	11.4±0.5(10–12)	11.5±0.5(11–12)	10.9±0.3(10–11)	11.1±0.5(10–12)	12.3±0.6(11–14)	12.7±1.1(12–17)	11.1±0.6(10–12)	11.1±0.7(10–12)	13.2±0.7(12–15)	13.2±0.8(12–15)	13±0.7(12–14)	12.5±0.611–14
Lateral line series scales	30.1±1.0(29–32)	29.6±1.1(28–32)	24.9±1.5(23–27)	26±1.4(23–27)	27.9±0.9(26–29)	27.1±1.3(25–29)	25.6±1.6(22–28)	25.4±1.5(23–29)	36.3±2.8(33–43)	37.1±2.7(33–43)	27.1±1.1(24–29)	27±1.2(24–29)
Caudal peduncle scales	11.6±0.6(10–13)	11.6±0.7(10–13)	10±0.5(9–11)	10.3±0.8(9–12)	9.9±0.8(8–11)	9.7±0.6(9–11)	9.26±0.6(8–11)	9.4±0.6(8–10)	12.6±1.1(10–14)	12.5±1.4(9–15)	9.2±0.7(8–11)	9.3±0.8(8–11)
Gill raker	9.2±0.5(8–10)	9.3±0.5(8–10)	10.8±0.5(10–12)	11.1±0.7(10–13)	10.7±0.7(9–12)	10.7±0.8(9–12)	10.9±0.7(9–13)	10.7±0.8(9–12)	9.7±0.1(8–12)	9.7±0.7(8–11)	9.8±0.6(8–11)	9.6±0.7(8–11)
Flank bars	15.9±1.4(12–19)	–	10.7±0.9(9–13)	–	11.9±1.5(8–15)	–	12.4±1.2(10–16)	–	11±1.2(8–13)	–	13.8± 1.7(11–17)	–

#### Comparative remarks.

*Aphanius arakensis* is close to the other Iranian *Aphanius* species in having a similar external morphology but differs by a high number of flank bars, 12–19, commonly, 15–16 (vs. 8–13, commonly, 11–12 in *Aphanius vladykovi*; 10–16, commonly, 12–13, in *Aphanius farsicus*; 8–15, commonly, 11–13 in *Aphanius sophiae*; 9–13, commonly, 10–11 in *Aphanius isfahanensis* and 11–17, commonly, 13–14 in *Aphanius pluristriatus*), otolith morphology and by having 19 molecular apomorphies in the cytochrome *b* gene. The new species (both males and females) can be further distinguished from *Aphanius vladykovi* by 28–32 lateral line series scales (vs. 33–43), and by less relative width and length of scales, 3.3–4.6 and 3.3–4.5% SL, respectively (vs. 1.9–3.2 and 1.9–3.3, respectively). It differs from *Aphanius sophiae* in having 10–13 caudal peduncle scales (vs. 8–11), less gill rakers numbers, 8–10 (vs. 9–12), and by a greater interorbital distance, 0.9–1.2% head width (vs. 0.8–1.1). The new species differs from *Aphanius farsicus* in having 6–8 pelvic fin rays (vs. 6–7), and by a smaller eye diameter, 10.7–14.8% preanal distance (vs. 10.6–17.0). It can be distinguished from *Aphanius isfahanensis* by 8–10 gill rakers (vs. 10–13), and by a shorter caudal peduncle, 29.0–34.8% preanal distance (vs. 27.4–38.4). It differs from *Aphanius pluristriatus* in having 10–13 caudal peduncle scales (vs. 8–11), 28–32 lateral line series scales (vs. 24–29) and by a smaller eye diameter, 10.7–14.8% preanal distance (vs. 12.4–18.1).

#### Distribution and habitat.

**Figure 4.**
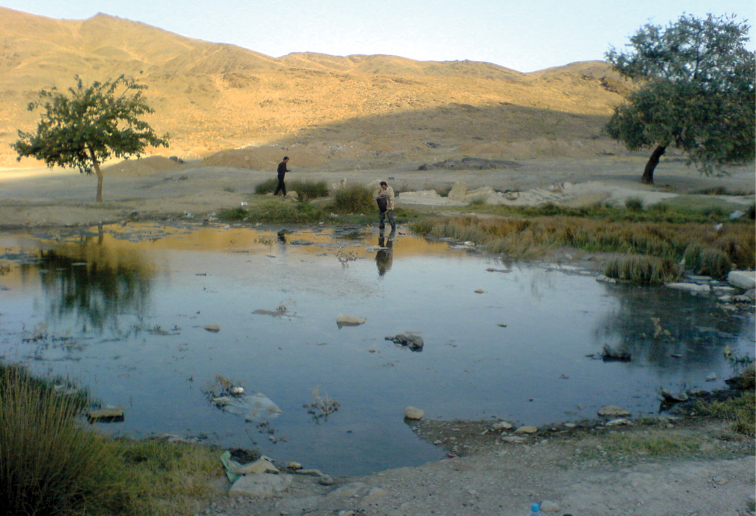
Natural shallow pond and type locality of *Aphanius arakensis* sp. n., in the Namak Lake Basin, 5 km SE of Arak city, Iran (see Fig. 1).

**Figure 5.**
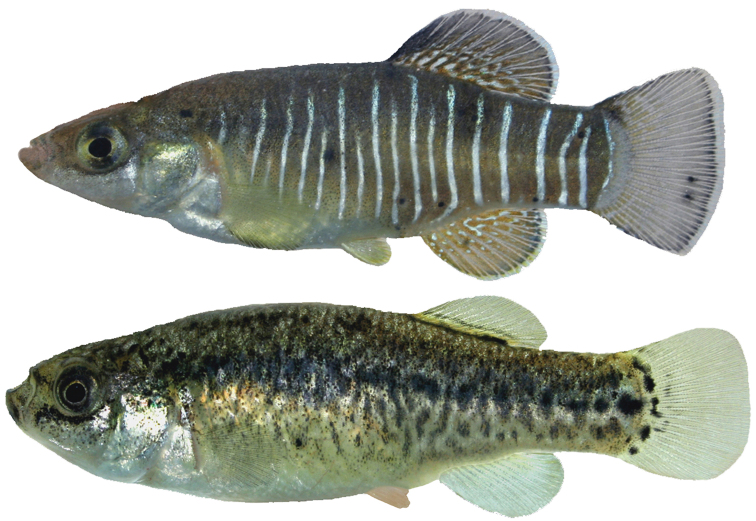
Male (above) and female specimens (not preserved) of *Aphanius arakensis* sp. n., collected from Cheshmeh Nazi (Nazi spring, 33°42'56.8"N, 50°04'21.9"E) near type locality, Namak Lake Basin.

The species has been collected from a small natural shallow pond (Fig. 4) in the Namak Lake basin, 5 km south east of the city of Arak (Fig. 1). This pond, which is about 6 x 4 m in size, is fed by the drainage of a nearby natural spring. During sampling, the water body was almost stagnant and water temperature was 23°C. There was no vegetation in the pond, but the surrounding area was covered with *Juncus* sp. and *Typha* sp. The bottom of the pond was generally muddy with small gravels. The habitat was in a bad condition due to anthropogenic pollution. Around collection time, the new *Aphanius* species was the only fish observed living in the pond. In addition, the new species can be found in several springs located in close proximity to the type locality (Fig. 5).

#### Etymology.

The species name refers to the city of Arak, which is located in close proximity to the type locality. Arak is the capital of the Markazi province in north-central Iran. A proposed common name is Arak tooth-carp. Farsi name is Kapour-e-dandandar-e-Arak.

##### Phylogenetic relationships

**Figure 6.**
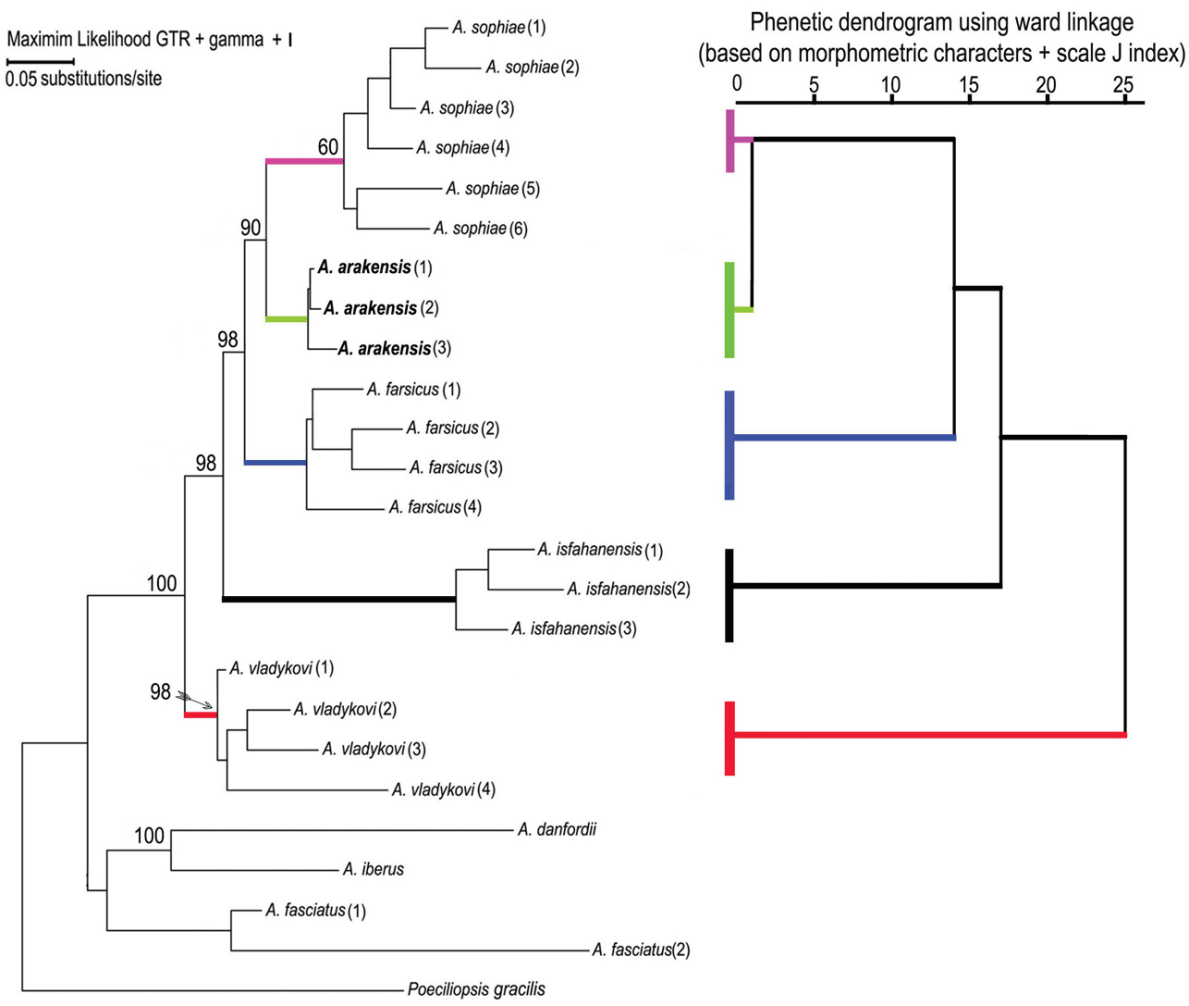
Phylogenetic relationships of *Aphanius arakensis* sp. n., and other endemic species of *Aphanius* in Iran as indicated by maximum likelihood (based on cytochrome b sequences) and phenetic (based on morphometric characters of fish specimens + J scale indices) analysis. Numbers above nodes represent maximum likelihood bootstrap values based on 2000 replicates. Species and locations correspond to those listed in the Material section.

The parameters for the maximum likelihood are ln(L) = –85.11.91237, gamma shape parameter of 1.000, proportion of invariant sites of 0.097 and parsimony = 1556. The maximum parsimony phylogeny has a CI of 0.462 and RI of 0.747. The initial tree for the maximum likelihood analysis was obtained by the BIONJ algorithm. The trees of the maximum likelihood and maximum parsimony phylogenies (Fig. 6) are not significantly different in topology (Templeton test, P > 0.05). They support the hypothesis that *Aphanius arakensis* diverged from the clade leading to the present-day *Aphanius sophiae* and is sister to this species. Moreover, *Aphanius farsicus* is sister to *Aphanius arakensis* + *Aphanius sophiae*; sister to these taxa is *Aphanius isfahanensis*, and sister to all previously mentioned species is *Aphanius vladykovi*. The same topology (Templeton test, P > 0.05) is observed for the tree of the Neighbor Joining (NJ) distance-based analysis. Table 4 shows the estimation of evolutionary divergence between the sequences of the new species and its relatives.

**Table 3.** Summary of diagnostic molecular characters that differentiate *Aphanius arakensis* sp. n., from other Iranian *Aphanius* species. Of the 19 molecular apomorphies, 17 are transitions and two are transversions. Numbers above characters indicate the character’s position in the complete molecular character matrix.

**Position**	**2**	**2**	**2**	**3**	**3**	**4**	**5**	**5**	**6**	**6**	**6**	**6**	**7**	**7**	**8**	**8**	**8**	**8**	**8**
	**0**	**7**	**9**	**3**	**9**	**1**	**2**	**4**	**0**	**1**	**2**	**2**	**4**	**9**	**1**	**3**	**6**	**7**	**8**
	**7**	**6**	**1**	**3**	**5**	**1**	**6**	**9**	**6**	**6**	**2**	**4**	**5**	**5**	**1**	**7**	**0**	**5**	**7**
*Aphanius arakensis*	G	T	C	T	G	C	A	G	T	A	A	G	G	T	G	T	T	G	C
*Aphanius isfahanensis*	A	C	T	C	A	G	G	A	A	G	G	A	A	C	A	C	C	A	T
*Aphanius sophiae*	A	C	T	C	A	G	G	A	A	G	G	A	A	C	A	C	C	A	T
*Aphanius farsicus*	A	C	T	C	A	G	G	A	A	G	G	A	A	C	A	C	C	A	A
*Aphanius vladykovi*	A	C	T	C	A	A	G	A	A	G	G	A	A	C	A	C	C	A	A

## Discussion

### Probable reasons for morphological similarities between endemic *Aphanius* species

Several endemic *Aphanius* species are known that are soundly circumscribed by genetic differentiation and specific otolith morphology (see below), whereas they differ only weakly (or only in multivariate space) with regard to morphometry and meristics. Examples are *Aphanius isfahanensis* from central Iran, *Aphanius sophiae* and *Aphanius farsicus* from southern Iran (Hrbek et al. 2006, this study); another example from the Mediterranean area is *Aphanius baeticus* from Spain (Doadrio et al. 2002). *Aphanius arakensis* sp. n., from the Namak Lake basin represents yet another example for a species that is difficult to distinguish from its relatives based on external characters (with the exception of the features mentioned above). It is likely that the overall morphological similarity between these taxa are a result of the similar habitats, in which the various endemic *Aphanius* species are thriving. Thus, common environmental variables may have acted as a stabilizing selection on morphological characters (see also Hrbek et al. 2006). This offers an explanation as to why speciation events in *Aphanius* have affected genetic characters, rather than morphology, and why rapid genetic diversification can occur with little morphological change in this taxon (see also Adams et al. 2009).

### Probable reasons for otolith differences between endemic *Aphanius* species

Otolith morphology is known to support the distinctive taxonomic state of several *Aphanius* species (Reichenbacher et al. 2007, 2009a-b). However, otolith morphology has not been used in previous studies on the endemic Iranian inland *Aphanius* species. Here we have compared the otoliths of *Aphanius vladykovi*, *Aphanius isfahanensis*, *Aphanius farsicus* and *Aphanius sophiae* (Fig. 3) to show that these species are clearly different with regard to otolith morphology. Also *Aphanius arakensis* shows clear divergence of its otolith morphology in comparison to the other inland *Aphanius* species, in particular with regard to the weakly pronounced antirostrum (Fig. 3W–Aa).

Notably, the otoliths of *Aphanius vladykovi* are most distinctive in comparison to those of the other studied species as they are characterized by a long ventral part, angular overall shape and long rostrum (Fig. 3R–V). This uniqueness of the *Aphanius vladykovi* otoliths corresponds well to our and previous phylogenetic analyses, which have established *Aphanius vladykovi* as being sister to all other Iranian inland species that diverged approximately 10 Ma ago (Hrbek et al. 2003). As a result, *Aphanius* likely has a higher rate of divergence in otolith morphology than in overall morphology. This difference in divergence rate may be related to the function of the otoliths as parts of the inner ear. In general, otoliths provide a mechanism for measuring motion and position of the head relative to gravity (Manley et al. 2004). However, it is quite important for a fish to know from where a sound is coming, so as to be able to distinguish between different sounds and pick out the biologically most relevant sounds (Popper et al. 2005). In addition, differences in otolith morphology are related to the balance and orientation of a fish (Popper et al. 2005). This means that differences in otolith morphology can reflect changes in intraspecific communication and behavior in fishes, that may have acted as evolutionary pressures.

### Role of coloration pattern (flank bar numbers) in *Aphanius* diversification

Coloration and flank bar numbers are significant characters for the identification of *Aphanius* species, in particular for the identification of male individuals. Among the allopatric Iranian *Aphanius* species, males of *Aphanius arakensis* have the largest number of flank bars, and flank bars are non-overlapping, whereas the number of flank bars is lowest in *Aphanius sophiae*. Also the flank bars of the central Anatolian *Aphanius* species vary in thickness and number between species (Hrbek et al. 2002). However, the mechanisms underlying male flank bar variation have not been studied. We hypothesize that flank bar patterns play an important role in sexual selection, and thus represent important factors in the evolutionary history and speciation of *Aphanius*.

Sexual selection has long been believed to promote species divergence among groups of animals (see Kraaijeveld et al. 2010 for a review). Sexual selection may facilitate speciation because it can cause rapid evolutionary diversification of male mating signals and female preferences (Boughman 2001). Divergence in these traits may then contribute to reproductive isolation.

Several studies indicate that fishes can adapt to variation in underwater light environments by changing their colour, most likely as a result of a more effective intraspecific communication (Boughman 2001, 2002; Fuller 2002; Seehausen et al. 2008). Adding support to this interpretation is provided by studies on cichlids from the Victoria Lake (Seehausen et al. 2008) and African elephant fishes (Leal and Losos 2010). These studies indicate that variation in male nuptial coloration due to specific light conditions in different environments can result in ecological, phenotypic, genetic and behavioral differentiation. Additionally, color contrast with the visual background was found to be more important for effective intraspecific communication than color brightness (Fuller 2002). Thus, our conclusion is that the specific male flank bar patterns in different *Aphanius* species may have evolved as a response to different light regimes prevalent in respective habitats for increasing contrast and optimizing intraspecific communication. It can therefore be suggested that sensory-driven speciation mighthave played a prominent role in *Aphanius* speciation.

**Figure 7.**
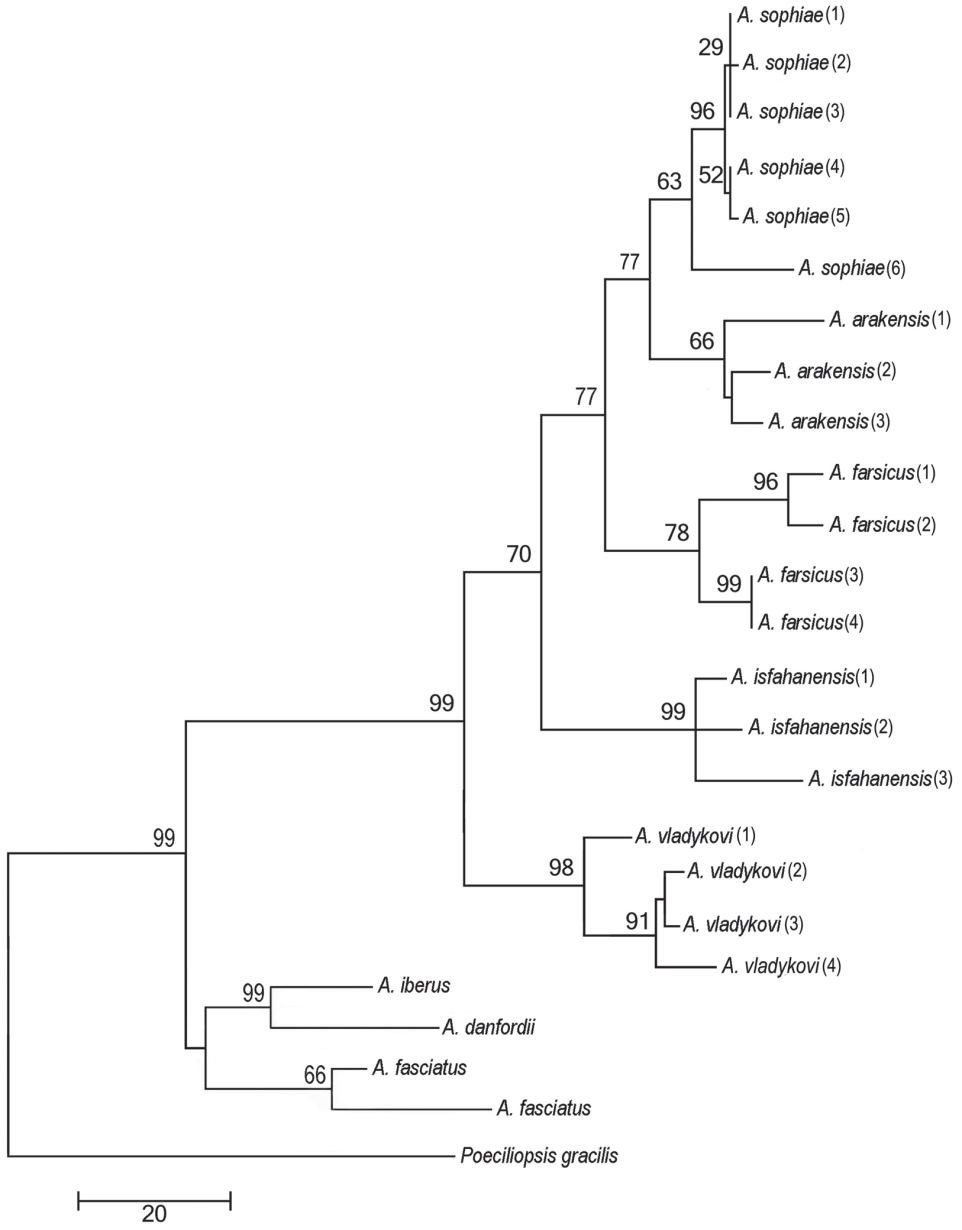
Phylogenetic relationships of *Aphanius arakensis* sp. n., and other endemic species of *Aphanius* in Iran as indicated by maximum parsimony (based on cytochrome b sequences) analysis. The maximum parsimony phylogeny has a CI of 0.462 and RI of 0.747. Numbers above nodes represent maximum parsimony bootstrap values based on 2000 replicates.

**Table 4.** Estimation of Genetic divergence (Kimura 2-parameter model) between the sequences of the *Aphanius arakensis* sp. n., and other Iranian *Aphanius* species. Aa = *Aphanius arakensis*, Ai = *Aphanius isfahanensis*, As = *Aphanius sophiae*, Af = *Aphanius farsicus* and Av = *Aphanius vladykovi*.

	**Aa1**	**Aa2**	**Aa3**	**Af1**	**Af2**	**Af3**	**Af4**	**Ai1**	**Ai2**	**Ai3**	**As1**	**As2**	**As3**	**As4**	**As5**	**As6**	**Av1**	**Av2**	**Av3**
**Aa1**																			
**Aa2**	0.000																		
**Aa3**	0.001	0.001																	
**Af1**	0.057	0.057	0.056																
**Af2**	0.054	0.054	0.053	0.009															
**Af3**	0.047	0.047	0.045	0.009	0.008														
**Af4**	0.047	0.047	0.045	0.011	0.007	0.001													
**Ai1**	0.119	0.119	0.118	0.116	0.115	0.113	0.114												
**Ai2**	0.098	0.098	0.096	0.092	0.093	0.090	0.092	0.025											
**Ai3**	0.113	0.113	0.111	0.111	0.109	0.105	0.106	0.012	0.019										
**As1**	0.041	0.041	0.040	0.068	0.066	0.058	0.060	0.118	0.097	0.112									
**As2**	0.029	0.029	0.027	0.053	0.051	0.042	0.044	0.105	0.084	0.099	0.015								
**As3**	0.036	0.036	0.034	0.054	0.051	0.050	0.051	0.106	0.085	0.100	0.018	0.007							
**As4**	0.029	0.029	0.027	0.053	0.051	0.042	0.044	0.105	0.084	0.099	0.015	0.000	0.007						
**As5**	0.030	0.030	0.029	0.051	0.053	0.044	0.045	0.107	0.084	0.100	0.016	0.001	0.008	0.001					
**As6**	0.033	0.033	0.031	0.054	0.052	0.047	0.048	0.108	0.087	0.102	0.018	0.004	0.008	0.004	0.005				
**Av1**	0.095	0.095	0.093	0.092	0.090	0.084	0.086	0.121	0.106	0.118	0.096	0.083	0.088	0.083	0.083	0.081			
**Av2**	0.082	0.082	0.081	0.077	0.073	0.075	0.076	0.115	0.098	0.112	0.086	0.070	0.073	0.070	0.072	0.075	0.027		
**Av3**	0.107	0.107	0.105	0.102	0.103	0.098	0.100	0.097	0.111	0.094	0.109	0.094	0.097	0.094	0.096	0.099	0.034	0.029	

## Conclusion

The noticeable features of the present-day diversity of the endemic *Aphanius* species in Iran include high genetic divergence and clear differences in otolith morphology, but only weak differences in general external morphology, morphometry and meristics. These patterns are probably caused by different rates of evolution in the mentioned characters that may be linked to the similarity of the individual environments, intra-species communication, and vicariance events. It is likely that additional *Aphanius* species are present in remote areas of Iran, especially in the Zagros and Alburz Mountains.

## Supplementary Material

XML Treatment for Aphanius arakensis
